# Efficacy of Minocycline in Acute Ischemic Stroke: A Systematic Review and Meta-Analysis of Rodent and Clinical Studies

**DOI:** 10.3389/fneur.2018.01103

**Published:** 2018-12-20

**Authors:** Zhaofu Sheng, Yang Liu, Hongmin Li, Wei Zheng, Bin Xia, Xin Zhang, V. Wee Yong, Mengzhou Xue

**Affiliations:** ^1^The Department of Cerebrovascular Diseases, The Second Affiliated Hospital of Zhengzhou University, Zhengzhou, China; ^2^The Henan Medical Key Laboratory of Translational Cerebrovascular Diseases, Zhengzhou, China; ^3^Hotchkiss Brain Institute, University of Calgary, Calgary, AB, Canada; ^4^Department of Clinical Neurosciences, University of Calgary, Calgary, AB, Canada

**Keywords:** meta-analysis, ischemic stroke, minocycline, clinical study, rodent study

## Abstract

**Objectives:** This study aimed to assess the efficacy of minocycline for the treatment of acute ischemic stroke.

**Background:** While there have been meta-analysis that surveyed the efficacy of minocycline in the treatment of acute stroke, they have some methodological limitations. We performed a new systematic review which was distinct from previous one by adding new outcomes and including new studies.

**Methods:** Document retrieval was executed through PubMed, Cochrane Central Register of Controlled Trials, the Stroke Center, NIH's Clinical Trials, Current Controlled Trials, and the WHO International Clinical Trials Registry Platform Search Portal before Jan 2018. The data meeting the inclusion criteria were extracted. Before meta-analysis, publication bias and heterogeneity of included studies were surveyed. Random and fixed-effects models were employed to calculate pooled estimates and 95% confidence intervals (CIs). Additionally, sensitivity and subgroup analyses were implemented.

**Result:** For clinical studies, 4 trials with 201 patients in the minocycline group, and 195 patients in the control group met the inclusion criteria; 3 were randomized trials. At the end of 90-day follow up or discharge day, results showed that the groups receiving minocycline were superior to the control group, with significant differences in the NIHSS scores (mean difference [MD], −2.75; 95% CI, −4.78, 0.27; *p* = 0.03) and mRS scores (MD, −0.98; 95% CI, −1.27, −0.69; *p* < 0.01), but not Barthel Index Score (MD, 9.04; 95% CI, −0.78, 18.07; *p* = 0.07). For rodent experiments, 14 studies were included. Neurological severity scores (NSS) was significantly improved (MD, −1.38; 95% CI, −1.64, −1.31; *p* < 0.01) and infarct volume was obviously reduced (Std mean difference [SMD], −2.38; 95% CI, −3.40, −1.36; *p* < 0.01) in the minocycline group. Heterogeneity among the studies was proved to exist for infarct volume (Chi^2^ = 116.12, *p* < 0.01; I^2^ = 0.89) but not for other variables.

**Conclusions:** Based on the results in our study, minocycline appears as an effective therapeutic option for acute ischemic stroke.

## Introduction

Stroke is a leading cause of death worldwide. In survivors, it can result in long-term disability; about 5 million stroke survivors are alive today (1). Although great advance has been obtained concerning stroke treatment, it still accounts for great mortality and morbidity worldwide (2).

As a semi-synthetic derivative of tetracycline, minocycline penetrates the blood-brain barrier (BBB) easily for its highly lipophilic property (3). Accumulated studies demonstrate its neurovascular protective effect in intracerebral hemorrhage and acute ischemia stroke patients (3–10). However, meta-analysis by Kohler et al. illustrated no obvious benefit of minocycline on modified Rankin Scale (mRS) ≤2 (7). There remains uncertainty regarding mRS reliability (11). Furthermore, the power of the mRS to present treatment effects is usually attenuated when the scale is dichotomized, discarding quantity details (12). By pooling mean differences (MD) of National Institute of Health Stroke Scale (NIHSS), Barthel Index (BI), and modified Rankin Scale (mRS) scores, Malhotra et al. demonstrated that minocycline may be a promising neuroprotective agent in acute stroke patients, especially in acute ischemia stroke subgroup (13).

In this study, we perform a new meta-analysis to evaluate the efficacy of minocycline for the management of acute ischemia stroke, specifically. Additionally, we also set out to investigate whether efficacy was different in animals exposed to minocycline using structural (infarct volume) and functional (neurobehavioral) outcomes in the rodent middle cerebral artery occlusion (MCAO) model.

## Methods

### Search Strategy

According to Preferred Reporting Items for Systematic Reviews and Meta-Analyses (PRISMA) guidelines, we performed this study. A systematic search was executed in PubMed, Cochrane Library. Additionally, four trial registry platforms were explored, including the World Health Organization (WHO) International Clinical Trials Registry Platform Search Portal (http://www.who.int/ictrp/en/), National Institutes of Health's Clinical Trials (http://clinicaltrials.gov/), the Stroke Center (http://www.strokecenter.org/trials/), Current Controlled Trials (http://www.controlled-trials.com/). We collected original articles published before Oct 2018. The keywords “minocycline” and “stroke” or “cerebral ischemia” were employed to search for related studies.

### Inclusion and Exclusion Criteria

For clinical trials, every observational or interventional study in English was taken into account for our research. The inclusion criteria were: adult patients (> 18 years old); trials comparing neuroprotective effects of minocycline vs. placebo in patients with acute ischemia stroke; each group with more than 5 patients; availability of clinical outcome data including NIHSS, mRS score, BI (Barthel Index). Studies with unclear or without extractable data were excluded.

For rodent experimental research, studies were included only when results of controlled comparisons of the effect of minocycline on the primary outcome measures in rodents subjected to focal cerebral ischemia were reported. We excluded studies using cellular/tissue models of ischemia. Studies were also excluded when the number of experimental animals could not be determined.

### Data Abstraction

Data collection was performed independently by the personnel and consensus were reached for any atypism.

For clinical trials, we extracted this information including overall study design, authors, locations, publication year, population characteristics, sample size (treatment group/control group), the time interval from stroke to therapy, dosage, duration of drug administration, delivery route, and outcome measures, follow-up period. Clinical end points included National Institutes of Health Stroke Scale (NIHSS) (14), modified Rankin Score (mRS) (15), and Barthel index (BI) (16). If multiple doses were employed, the sum of all doses administered was taken into account. If the functional outcomes were presented in more than 1 time points, we only employed the last time of evaluation.

For rodent experimental research, data coding included reference identification (authors, year of publication), nature of animals (species/strain, sex), stroke model (timing of intervention, duration of ischemia), minocycline administration information (dose, the timing of administration), and the time of outcome measurement. To assess the effectiveness of minocycline, indicators including neurological severity scores (NSS) and infarct volume were used to assess therapeutic effectiveness.

### Quality Assessment

Quality assessment was executed by 2 independent authors separately.

Risk bias of RCTs was evaluated by means of the Cochrane Collaboration tool for constituted by seven items, including random sequence generation, allocation concealment, blinding of participants and personnel, blinding of outcome assessment, incomplete outcome data, selective reporting, and other bias, which were assigned as low, unclear, or high (17). The risk of bias was plotted using the Review Manager 5.2 software. The methodological index for nonrandomized studies (MINORS) scale was employed to evaluate non-RCTs with scores ranging from 0 to 24 (18). The publication bias was not assessed because standard analytical techniques (Funnel plot, Egger's test) are not recommended if <5 studies are being analyzed (19).

Methodological quality of individual rodent experimental research was assessed according to published criteria (20, 21). These criteria were comprised of 10 domains, including peer-reviewed publication, statement of control of temperature, random allocation to treatment or control, blinded induction of ischemia, blinded assessment of outcome, use of anesthetic without significant intrinsic neuroprotective activity, appropriate animal model (aged, diabetic, or hypertensive), sample size calculation, compliance with animal welfare regulations, and statement of potential conflict of interests. Each study was given a quality score out of a possible total of 10 points. To identify publication bias, a funnel plot and Egger's test were performed to analyze variable, where data were available from more than 5 studies.

### Heterogeneity Assessment

Heterogeneity test was executed by means of Cochran's Q-test and the Higgins I^2^-test. A Cochran's Q *p* < 0.10 and I^2^ > 50% were deemed as significant heterogeneity (22). When significant heterogeneity was absent, the fixed-effects model was employed; otherwise, the random-effects model was used (23). For clinical studies, subgroup analyses were not available because trials with enough valuable data were not sufficient. Alternatively, sensitivity analysis through the leave-one-out approach to exclude abnormal results if the significant heterogeneity is available (*p* < 0.1 and I^2^ > 50%) (19).

### Statistical Analysis

The pooled outcome difference between minocycline group and control group were presented as mean difference (MD) or standardized mean difference (SMD) and 95% confidence interval (CI). Analyses were performed using Stata 12.0 (Stata Corp LP, College Station, TX, USA) and Review Manager 5.3 (The Nordic Cochrane Center, The Cochrane Collaboration, 2014, Copenhagen, Denmark).

## Results

### Search Results

**Figure 1:**
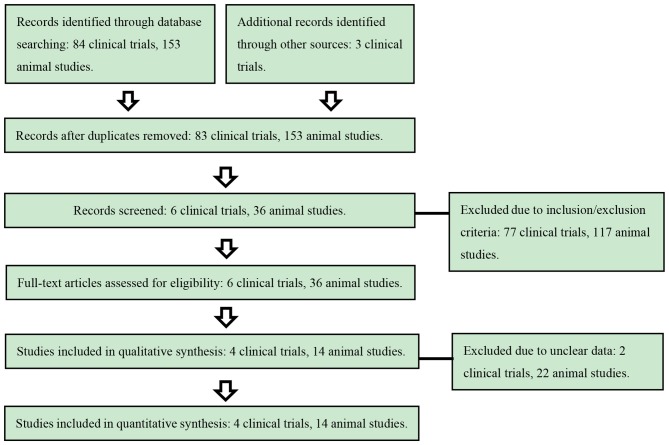
Flow chart of literature assessment for studies.

The initial screening excluded 81 clinical studies and 117 animal studies, leaving 6 clinical trials and 36 rodent experimental researches for full text assessment. Finally, 4 clinical trials and 14 rodent experimental researches were included (Figure 1).

### Study Characteristics

The characteristics of included clinical trials are presented in Table 1. Three hundred and ninety-six participants in 4 trials were included. Administration dosage of minocycline varied in each study, and the duration of treatment was 2.5 days (7), 3 days (5), or 5 days (6, 8), separately. Control groups were subject to placebo or conventional management. Follow up period was 3 months in 3 articles (6–8). Included trials were conducted in Australia, USA, Israel, and India. Additionally, Baseline outcomes of included clinical studies were illustrated in Table 2 and the significant difference was not observed between minocycline group and control group.

**Table 1:** Characteristics of included clinical studies.

**Study**	**Country**	**No**		**Mean age**		**Time interval from stroke to therapy**	**Method of administration**	**Follow-up (month)**	**Study design**
		**M**	**C**	**M**	**C**			
Kohler et al. (7)	Australia	47	48	67.7	67.9	Within 24 h	100 mg, IV, 12 hourly, 5 doses	3	Multicenter, prospective randomized open-label blinded study
Lampl et al. (6)	Israel	74	77	67.2	66.2	During 6–24 h	200 mg, PO, once daily, 5 days	3	Single center, randomized open-label, evaluator-blinded study
Padma Srivastava et al. (8)	India	23	27	52	57	During 6-24 h	200 mg, PO, once daily, 5 days	3	Single center, randomized single- blinded open-label study
Switzer et al. (5)	USA	57	43	65	61.8	Within 6 h	4 dose tiers (3.0, 4.5, 6.0, 10.0 mg/kg), IV, 12 hourly, 3 d	–	Single center, nonrandomized, dose- escalation trial

*M, minocycline group; C, control group; IV, intravenously; PO, per orally*.

**Table 2:** Baseline outcomes of included clinical studies.

**Study**	**Baseline NIHSS**		**Baseline mRS**		**Baseline BI**	
	**M**	**C**	**M**	**C**	**M**	**C**
Kohler et al. (7)	9.1	8.7	–	–	–	–
Lampl et al. (6)	7.5	7.6	2.8	2.9	70	63.9
Padma Srivastava et al. (8)	12.3	11.5	3.7	3.7	42.8	40.3
Switzer et al. (5)	8.7	9.5	–	–	–	–

*NIHSS, National Institutes of Health Stroke Scale; mRS, Modified Rankin Scale; BI, Barthel Index; M, minocycline group; C, control group*.

The characteristics of included rodent experimental studies are summarized in Table 3. We retrieved 14 publications, which included 6 comparisons of neurological function scores (137 treated, 142 control) and 14 comparisons of infarct volume (91 treated, 83 control). Within these publications, 1 reported both IV and IP (34), 6 IV only (26, 27, 31–33, 36), and 5 IP only (25, 28, 30, 35, 37), 1 PO only (24), 1 not mentioned (29).

**Table 3:** Characteristics of included rodent experimental studies.

**Study**	**Animals (Male)**	**Stroke model**	**Minocycline administration**	**Dosage and routine**	**Neurological function assessment**	**Infarct volume measurement**
Bhatt and Addepalli (24)	Wistar rats	Filament occlusion of the MCA for 2 h	3-week treatment after 1 h ischemia and 24 h reperfusion injury	50 mg/kg, IG	–	After 24 h of reperfusion
Hayakawa et al. (25)	DDY mice	Filament occlusion of the MCA for 4 h	1 day after occlusion for 14 days, once daily	10 mg/kg, IP	14 days after cerebral ischemia	24 h after ischemia
Hoda et al. (26)	C57BL/6J mice	Embolizing pre-formed clot into the MCA	Immediately after clot injection	6 mg/kg, IV	24 h post stroke	24 h after stroke
Jin et al. (27)	Sprague-Dawley rats	Filament occlusion of the MCA for 90 min	15 min after reperfusion onset	3 mg/kg, IV	–	The end of 48 h of reperfusion
Jin et al. (28)	C57/BL6 mice	Filament occlusion of the MCA for 2 h	2 h after stroke, twice a day	90 mg/kg, IP	8 h after reperfusion	After 2 h of ischemia and 48 h of reperfusion
Li and McCullough (29)	C57/B6 mice	Filament occlusion of the MCA for 90 min	Two doses every 12 h, the first dose 30 min after the onset of ischemia	45 mg/kg	–	24 h after stroke
Li et al. (30)	Sprague-Dawley rats	Filament occlusion of the MCA for 60 min	6 h after reperfusion using the same dose for 4 weeks, once daily	50 mg/kg for 1 week followed by 25 mg/kg for the remaining 3 weeks, IP	–	4 weeks after stroke
Martín et al. (31)	Sprague-Dawley rats	Filament occlusion of the MCA for 2 h	1 h after ischemia and a daily dose for the following 6 days	10 mg/kg, IV	–	7 days after reperfusion
Matsukawa et al. (32)	Sprague-Dawley rats	Filament occlusion of the MCA for 30 min	60 min after the reperfusion, a single bolus	20 mg/kg, IV	72 h post stroke just prior to euthanasia	72 h post stroke
Murata et al. (33)	Spontaneously hypertensive rats	Embolizing pre-formed clot into the MCA	4 h after ischemia	3 mg/kg, IV	–	24 h after ischemia
Soliman et al. (34)	Wistar rats	Filament occlusion of the MCA for 90 min	Immediately after reperfusion onset and the following 7 days	20 mg/kg IV for the first dose and IP for the following doses	–	Day 14 post stroke
Wang et al. (35)	Wistar rats	Embolizing pre-formed clot into the MCA	1 and 4 h after embolization on the first day, and 24 and 32 h on the second day	45 mg/kg on the first day, 22.5 mg/kg on the second day, IP	–	48 h after embolization
Xu et al. (36)	Sprague-Dawley rats	Filament occlusion of the MCA for 90 min	4, 8, and 12 h post stroke	10 mg/kg, IV	4 h post stroke	24 h post stroke
Yenari et al. (37)	C57/BL6 mice	Filament occlusion of the MCA for 2 h	30 min and 12 h after ischemia, 2 doses	45 mg/kg, IP	24 h after ischemia onset	24 h post stroke

*MCA, middle cerebral artery; IG, intragastrically; IP, intraperitoneally; IV, intravenously*.

### Quality Assessments

**Figure 2:**
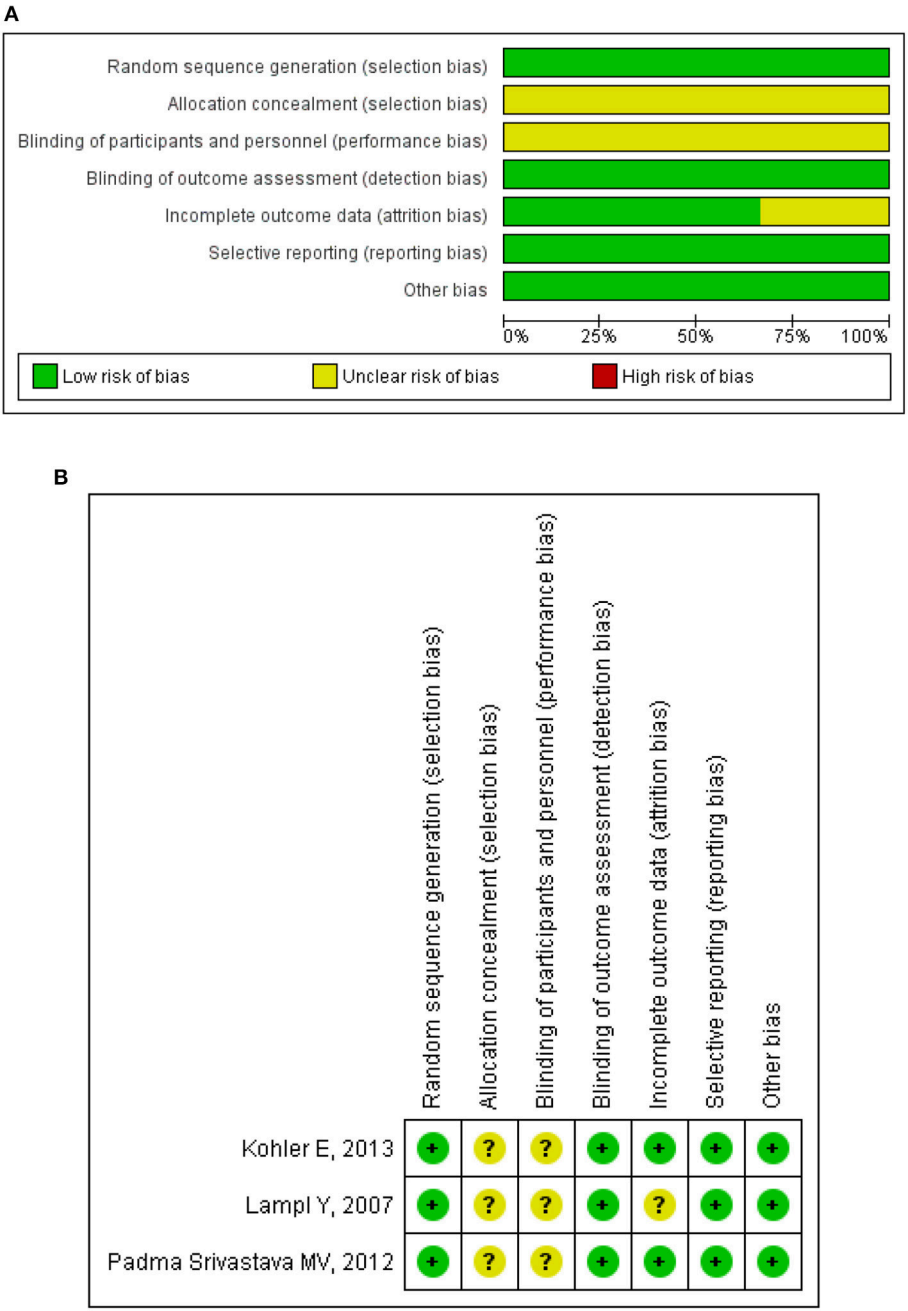
Methodological quality assessment for randomized clinical trials. **(A)** Risk of bias graph: review judgments about each risk of bias item presented as percentages across all included studies. **(B)** Risk of bias summary: review judgments about each risk of bias item for each included study. “+,” low risk of bias; “–,” high risk of bias; “?,” unclear risk of bias.

For RCT studies, risk bias of allocation concealment and blinding was high in 3 clinical studies (6–8). In addition, 2 clinical studies reported the follow-up rates as 92.6% (7) and 81.8% (8), separately, which were not mentioned in another clinical study (6). Except for these 3 items, no high risk of bias was observed in any of the other items (Figure 2). For non-RCT study (5), quality assessment results showed a moderate risk of bias (Table 4).

**Table 4:** Methodological quality of the nonrandomized controlled clinical trials.

**Quality assessment for nonrandomized trials**	**Kohler et al. (7)**
A clearly stated aim	2
Inclusion of consecutive patients	2
Prospective data collection	0
Endpoints appropriate to the aim of the study	2
Unbiased assessment of the study endpoint	0
A follow-up period appropriate to the aims of study	0
Less than 5% loss to follow-up	0
Prospective calculation of the sample size	0
An adequate control group	1
Contemporary groups	0
Baseline equivalence of groups	1
Adequate statistical analyses	2
Total score	10

*The items are scored 0 (not reported), 1 (reported but inadequate) or 2 (reported and adequate). The global ideal score being 16 for non-comparative studies and 24 for comparative studies*.

**Figure 3:**
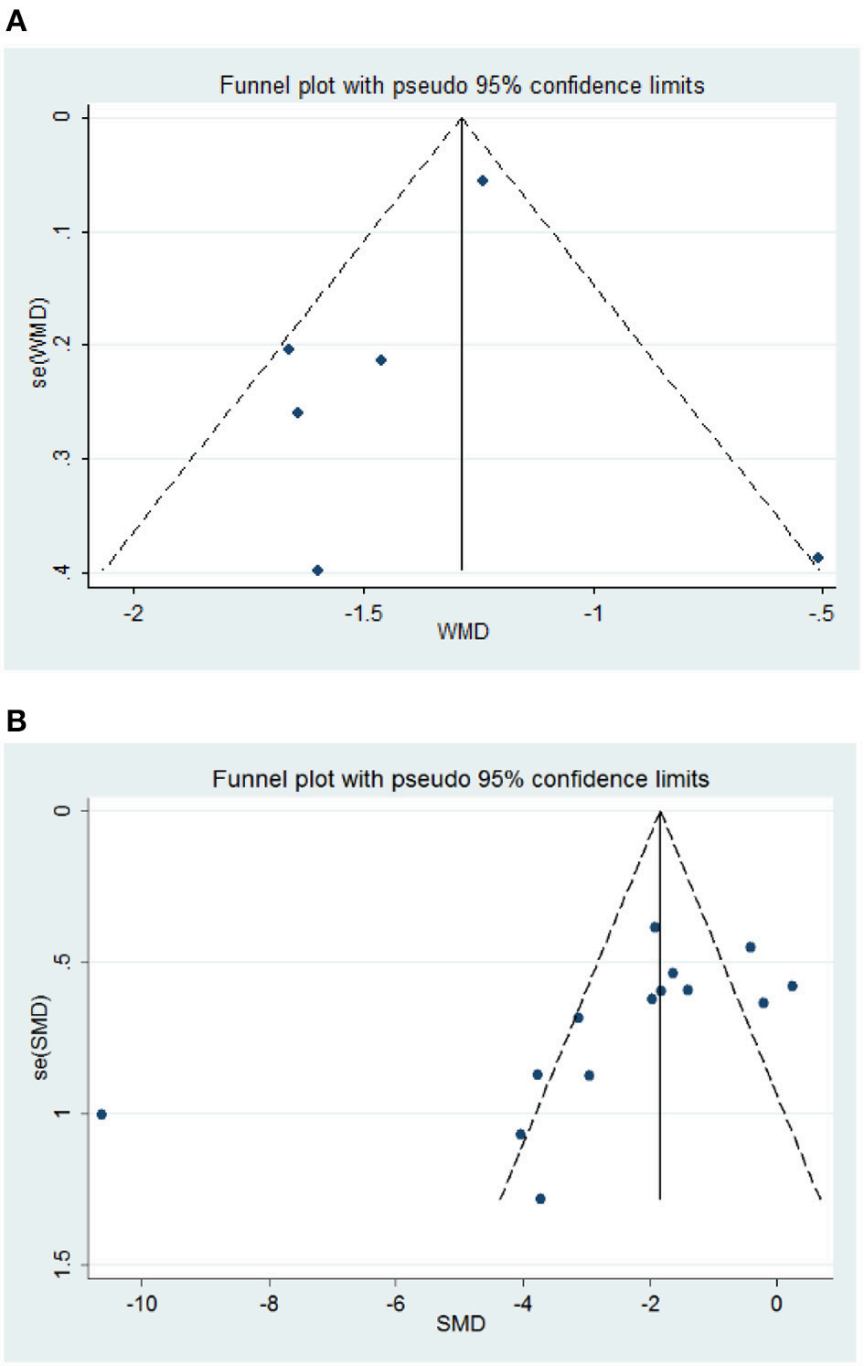
Funnel plot assessing publication bias of rodent experimental studies. **(A)** Neurological Severity Score, NSS. **(B)** Infarct volume. Vertical lines represent the summary effects; dashed lines denote the 95% CIs.

The overall methodological quality of rodent studies is summarized in Table 5, where on the 10 items, the median score was 5.8 (first, third quartiles: 5, 7, respectively; range, 3–8). Studies were compliant with the majority of the items; exceptions were “blinded allocation to ischemia” (0%, not mentioned in all included studies), “random allocation to treatment or control” (35.7%, not mentioned in 9 included studies), “blinded assessment of outcome” (35.7%, not mentioned in 9 included studies), and use of anesthetic without significant intrinsic neuroprotective activity (7.14%, not mentioned in 2 included studies). The funnel plot of neurological improvement suggested no publication bias (Egger's test intercept: −0.74, *p* = 0.48; Figure 3A). However, the funnel plots of infarct volume were hints of potential publication bias, which was also demonstrated by the Egger's test (intercept: −5.82, *p* = 0.025; Figure 3B).

**Table 5:** Methodological quality of the rodent experimental studies.

**Quality category**	**No. of studies with quality**
Peer reviewed publication	14
Control of temperature	10
Random allocation to treatment or control	5
Blinded induction of ischemia	0
Blinded assessment of outcome	5
Use of anesthetic without significant intrinsic neuroprotective activity	1
Animal model (aged, diabetic, or hypertensive)	14
Sample size calculation	14
Compliance with animal welfare regulations	10
Statement of potential conflict of interests	9

*The scale used was originally proposed by Macleod et al. with a possible maximum score of 10. One point is given for each criterion met*.

### Intervention Effects in Clinical Trials

#### NIHSS Scores

**Figure 4:**
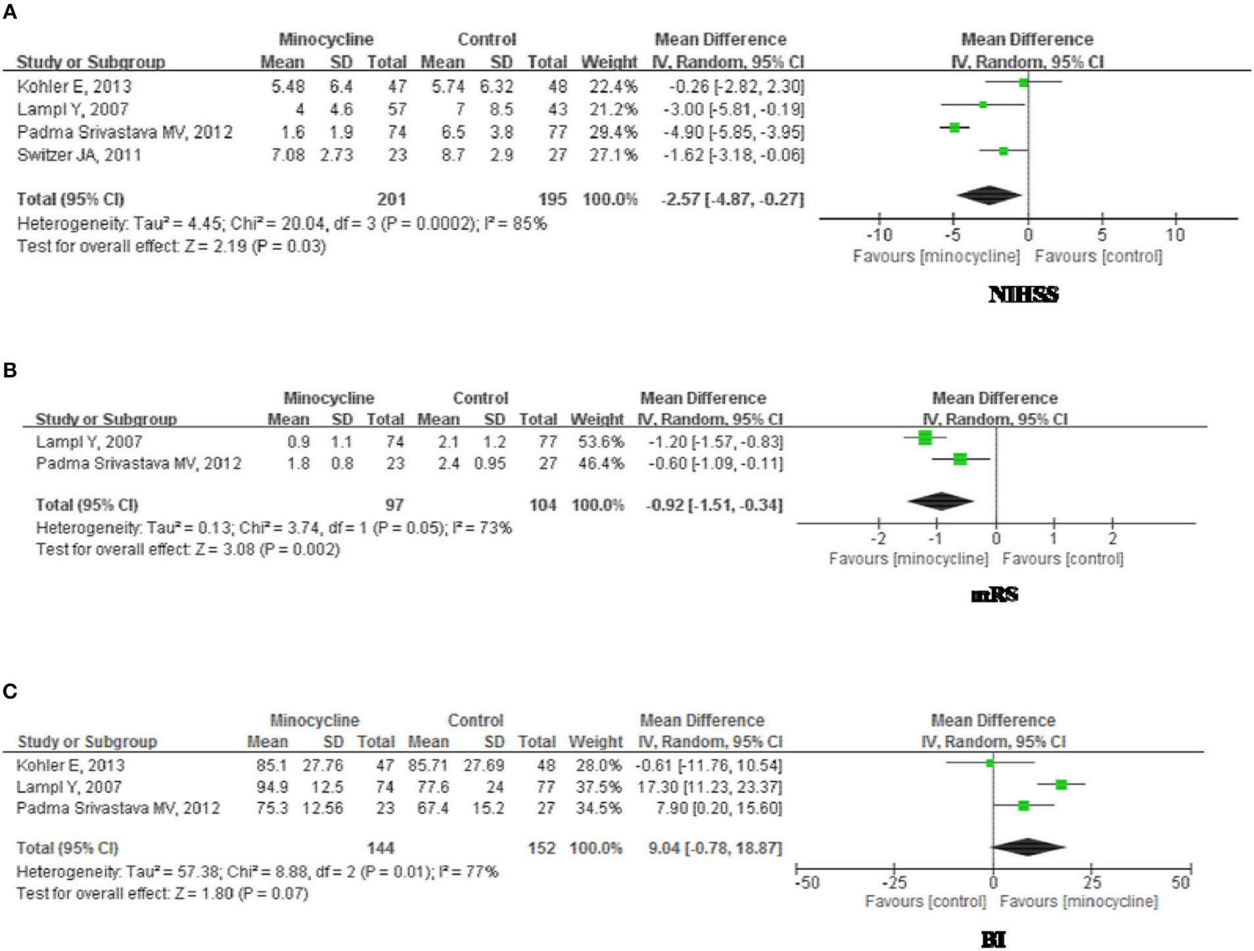
Forest plot illustrating the meta-analysis of the clinical outcome. **(A)** National Institutes of Health Stroke Scale, NIHSS. **(B)** modified Rankin Scale, mRS. **(C)** Barthel Index, BI.

NIHSS scores were presented over for 396 subjects in 4 studies. NIHSS scores (Figure 4A) were significantly different between groups (MD, −2.75; 95% CI, −4.78, 0.27; *p* = 0.03) during 3 months follow up period. There was significant heterogeneity among the studies (Chi^2^ = 20.04, df = 3, I^2^ = 85%, *p* = 0.0002); therefore, a random-effects model was used. Sensitivity analysis of the NIHSS scores suggested that the direction and magnitude of the combined estimates remains the same (Table 6). However, the exclusion of individual research (6) led to a pooled estimate of NIHSS scores of −2.42 (95% CI: −5.03, 0.46) but not statistically significant (*p* = 0.10), and contributed to greater study heterogeneity (Chi^2^ = 19.84, *p* < 0.0001; I^2^ = 90%). In addition, the exclusion of another study (8) led to a pooled estimate of NIHSS scores of −1.57 (95% CI: −2.78, −0.36) which was more statistically significant (*p* = 0.01) and no study heterogeneity (*p* = 0.37; I^2^ = 0%).

**Table 6:** The results of sensitivity analysis of clinical trials.

**Study omitted**	**Estimate**	**[95% Conf. interval]**	
**NIHSS**			
Switzer et al. (5)	−2.42	−5.30	0.46
Padma Srivastava et al. (8)	−2.89	−5.81	0.03
Lampl et al. (6)	−1.57	−2.78	−0.36
Kohler et al. (7)	−3.25	−5.63	−0.87
**BI**			
Padma Srivastava et al. (8)	8.95	−8.60	26.51
Lampl et al. (6)	4.61	−3.55	12.77
Kohler et al. (7)	12.91	3.72	22.10

*NIHSS, National Institutes of Health Stroke Scale; BI: Barthel Index*.

#### mRS Scores

mRS scores were illustrated for 201 subjects in 2 studies. There was significant heterogeneity among the trials (Chi^2^ = 3.74, df = 1, *P* = 0.05; I^2^ = 0.73); therefore, a random-effects model was employed. At 3 months follow up, mRs scores (Figure 4B) of minocycline group were greater than that of control group (MD, −0.98; 95% CI, −1.27, −0.69; *p* < 0.01).

#### BI Scores

BI scores were presented for 296 subjects in 3 studies. There was significant heterogeneity among the studies (Chi^2^ = 8.88, df = 2, *P* = 0.01; I^2^ = 0.77); therefore, a random-effects model was used. At 3 months follow up, there were no significant differentiation between groups (MD, 9.04; 95% CI, −0.78, 18.07; *p* = 0.07; Figure 4C). Sensitivity analysis (Table 6) showed that omitting individual research (7) resulted in a pooled estimate of BI of 12.91 (95% CI: 3.72, 22.10), which was statistically significant (*p* = 0.006). Elimination of this study brought about heterogeneity (*p* = 0.06; I^2^ = 72%).

### Intervention Effects in Rodent Experiments

#### NSS

**Figure 5:**
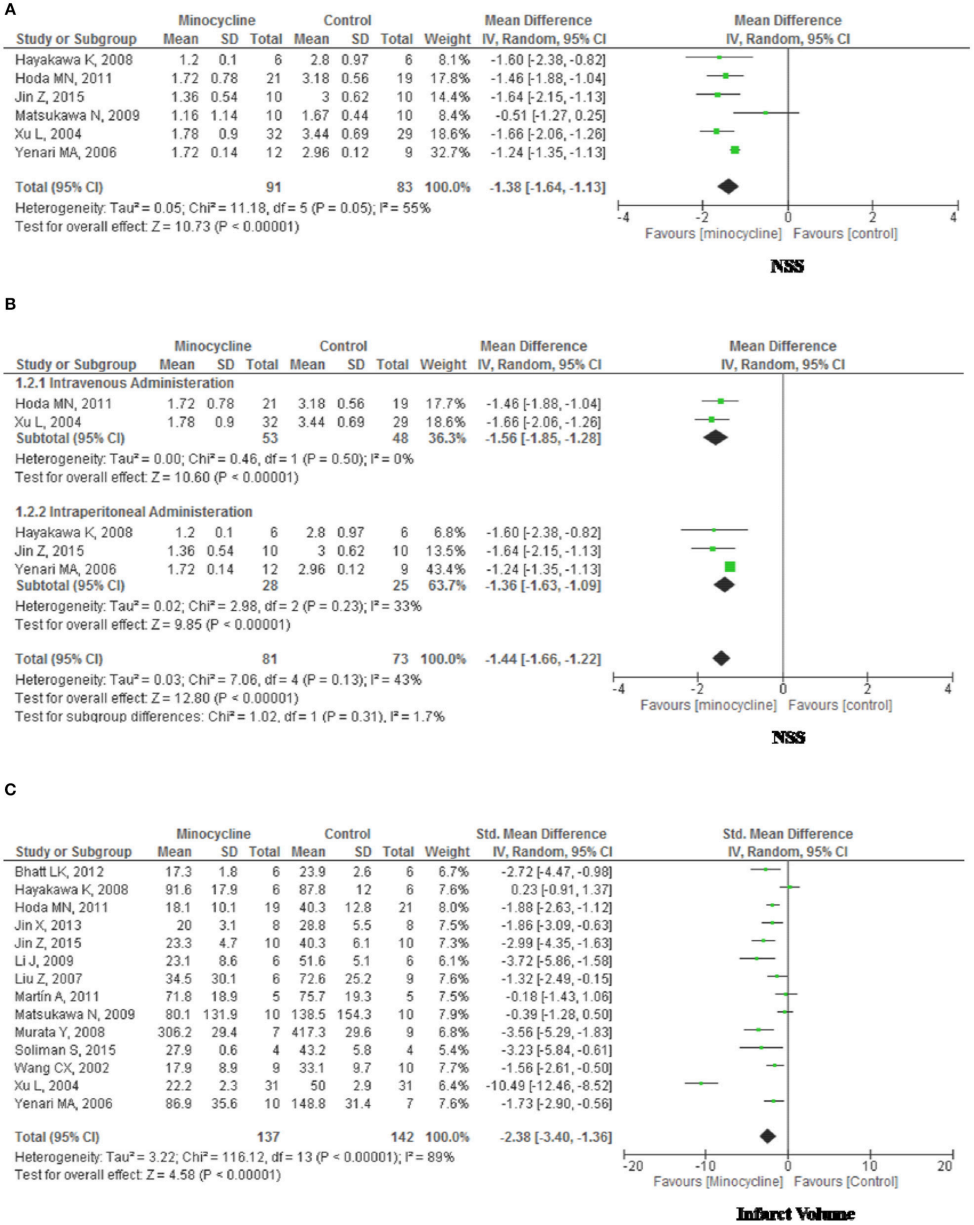
Forest plot illustrating the meta-analysis of the rodent experimental outcome. **(A)** Neurological Severity Score, NSS. **(B)** NSS stratified by administration route. **(C)** Infarct volume.

There was moderate heterogeneity among the studies (Chi^2^ = 11.18, df = 5, *P* = 0.05; I^2^ = 0.55); therefore, a random-effects model was used. NSS scores between two groups were significantly different (MD, −1.38; 95% CI, −1.64, −1.31; *p* < 0.01). In the subgroup analysis stratified by administration route, functional outcome (Figures 5A,B) was significantly improved in minocycline group (Intravenous administration: MD, −1.56; 95% CI, −1.85, −1.28; *p* < 0.01. Intraperitoneal administration: MD, −1.36; 95% CI, −1.63, −1.09; *p* < 0.01). Furthermore, subgroup or overall analysis indicated no significant between-study heterogeneity.

#### Infarct Volume

There was significant heterogeneity among the studies (Chi^2^ = 116.12, df = 13, *p* < 0.01; I^2^ = 0.89); therefore, a random-effects model was used. Infarct Volume (Figure 5C) were significantly different between groups (Std mean difference [SMD], −2.38; 95% CI, −3.40, −1.36; *p* < 0.01). In the subgroup analysis stratified by species, administration route, experimental stroke model and format of outcomes, separately, we were not able to identify covariates other than study quality that could explain the observed heterogeneity (data not shown).

## Discussions

Accumulated evidence indicates that minocycline may be a potential therapy for patients with stroke (3, 38). In this study, we included 4 most recent clinical research and 14 rodent experimental studies for meta-analysis and demonstrated the efficacy of minocycline in improving functional outcomes. Neuroprotective activities of minocycline may attribute to miscellaneous function, including anti-oxidative, anti-inflammatory, anti-apoptotic effects, and inhibition of glutamate toxicity (3, 39, 40). It also acts as matrix metalloproteinase-9 (MMP-9) inhibitor. MMP-9 may mediate tissue injury caused by human ischemic stroke and links with intracranial hemorrhage transformation due to thrombolytic therapy (41). In addition, minocycline attenuates brain swelling and BBB disruption after intracerebral hemorrhage via an iron-chelation mechanism (42). Based on the fact that the half-life of minocycline is about 24-h, every 24 h dosing is appropriate (43). Moreover, several studies support the safety of minocycline in acute stroke (41). All these make it possible to become a potential stroke treatment.

The NIHSS is a typical tool for pre-randomization and post-treatment assessment in the clinical trial and may be completed in 10 min. It provides a reliable, reproducible, and validated measure of stroke severity (44). Despite its inherent ceiling and floor effects (45), BI has the advantages of being simple and quick to complete (46). Furthermore, BI differentiated disability better in lower than higher disability (47). Thus, Barthel index as a standard outcome measure is still appropriate for long run follow up. Compared to the BI, the mRS seems to reveal small but significant treatment effect changes in mild to moderate stroke patients (45). In this study, NIHSS, BI, and mRS were employed to justify the effects of minocycline in stroke. A specific cut-off or alterable score to present an important end point is somewhat ambiguous (48). In the simple dichotomous approach, the scores come down to just two states, and it may be difficult to determine the optimal point for dichotomization (12). Thus, in our study the use of mean scores rather that dichotomizing the scales was favored.

For statistic clinical heterogeneity, the main limitations of our study lie in the random error caused by the relatively small sample size. Furthermore, as the sample of patients was small, it was not possible to isolate subgroups of patients with a better outcome, according to different administration routes, etc. Meta-analyses for continuous outcomes showed higher I^2^ than that of binary outcomes (49). Furthermore, I^2^ get a substantial bias when the research quantity is small, and the positive bias may exists when the fraction of heterogeneity is small (50). Methodological differences in 4 trials could not be neglected. The predictable validity of NIHSS scores for arterial occlusion is time-dependent, decreasing from symptom onset to clinical evaluation (51). In our study, the time-point for the NIHSS evaluation were varied in different trials included. At 6 months post-stroke, the maximum sensitivity of mRS in differentiating rehabilitation is achieved (15). However, no more than 3-month of clinical follow-up was performed in 4 trials. In addition, there are the potential for systematic error in 3 open-label allocation trials (6, 7). RCTs and non-RCT synergistically provide more and better information about superior of alternative treatments (8, 52, 53). The potential of selection bias may constitute low internal validity in the nonrandomized trial. All clinical studies included were lack of adjustment on baseline differences (54).

Because substantial heterogeneities existed in all clinical outcomes, we adjusted those heterogeneities and potential publication bias by sensitivity analysis. In our present study, estimates of pooled NIHSS scores and its heterogeneity may be affected by the sequential exclusion of particular trial. Even though benefits of minocycline on BI was presented after omitting outliers for sensitivity analysis, evidence supporting improvement effect of minocycline on BI is insufficient. All these mentioned above indicating that analysis of clinical results in our present may be influenced by each study.

In rodent studies, infarct size appeared to be a relatively objective outcome. Methodological quality varied in each rodent experimental research. Using stratified meta-analysis models, we were not able to identify covariates other than study quality that could explain the observed heterogeneity in infarct volume. It was assumed that the methodological variables and small sample size of included rodent studies contribute to significant heterogeneity synergistically. Additionally, most animal stroke models require anesthesia and some anesthetic agents, including isoflurane, exhibits neuroprotective properties and may improve neurological deficits due to brain injuries (55), and these anesthetics should be avoided in animal stroke model. Furthermore, the performance of neurobehavioral scores should be evaluated in future studies (21).

## Conclusions

The animal data are consistent with the available clinical data suggesting a role for minocycline to facilitate recovery of function. Much larger randomized studies and animal research are necessary for confirmation of the results.

## Author Contributions

This manuscript has been read and approved by all authors for publication. ZS contributed to study design, YL contributed to part of the writing, HL contributed to data analysis, WZ contributed to part of the writing, BX contributed to part of the writing, XZ contributed to collect and record data, VY contributed to part of the writing and MX contributed to further improvement of the writing.

### Conflict of Interest Statement

The authors declare that the research was conducted in the absence of any commercial or financial relationships that could be construed as a potential conflict of interest.

## Abbreviations

NIH: National Institutes of Health
WHO: World Health Organization
BBB: Brain-blood barrier
mRS: modified Rankin Scale
MCAO: Middle cerebral artery occlusion
PRISMA: Preferred Reporting Items for Systematic Reviews and Meta-Analyses
NIHSS: NIH Stroke Scale
BI: Barthel Index
NSS: Neurological severity scores
RCT: Randomized controlled trials
MINORS: methodological index for nonrandomized studies
MD: mean difference
SMD: standardized mean difference
CI: 95% confidence interval
IP: intraperitoneal
IV: intravenous
PO: peros.
